# Minority Stress and Psychosocial Influences on Cognitive Performance in Sexual Minority Older Adults

**DOI:** 10.1093/geroni/igad110

**Published:** 2023-09-24

**Authors:** Riccardo Manca, Annalena Venneri

**Affiliations:** Department of Life Sciences, Brunel University London, Uxbridge, UK; Department of Medicine and Surgery, University of Parma, Parma, Italy; Department of Life Sciences, Brunel University London, Uxbridge, UK; Department of Medicine and Surgery, University of Parma, Parma, Italy

**Keywords:** Cognition, Marital status, Mental health, Sexual orientation

## Abstract

**Background and Objectives:**

Sexual minorities experience health inequalities, but little is known about differences in neurocognitive health between heterosexual and sexual minority older adults and potential risk factors. To investigate minority stress, depression, and marital status as risk factors for worse cognitive performance in sexual minority older adults.

**Research Design and Methods:**

A total of 336 sexual minorities and 5,561 heterosexual participants aged 50+, noninstitutionalized, and free from neurodegenerative diseases from Wave 6 of the English Longitudinal Study of Ageing were included. Cognitive performance (i.e., temporal orientation, episodic memory, and fluid intelligence) of sexual minority and heterosexual older adults was compared using general linear models including age, sex, and education as covariates. The differential impact of minority stress, depressive symptoms, and marital status on cognition in the 2 groups were also tested. Analyses were weighted for sampling probability and differential nonresponse.

**Results:**

Sexual minority participants were more likely to report minority stress and to be single but had better episodic memory than heterosexual participants. Depression and being single were associated with worse cognitive performance in both groups. However, minority stress was negatively associated (*B* = −2.116, *p* = .016) with fluid intelligence in the sexual minority group only.

**Discussion and Implications:**

Better memory in sexual minority participants and a negative effect of risk factors on cognition are in line with previous studies. However, this study provides the first evidence of a potential negative impact of minority stress on cognitive performance in sexual minorities. Further investigations are needed to assess minority stress more in detail and clarify its potential mechanisms of action on cognition in sexual minorities.

**Translational Significance:** This study investigated, for the first time, a proxy measure of minority stress (i.e., self-reported negative social experiences due to sexual orientation) as a predictor of cognitive performance in a large sample of sexual minority older adults. Along with other established risk factors (i.e., depression and being single), minority stress was also associated with worse cognitive performance on a test of fluid intelligence in sexual minority older adults only. This finding suggests that the assessment of minority stress-related factors may offer useful insights for the clinical management of cognition of sexual minority older adults.

## Background and Objectives

Neurocognitive aging is a process characterized by great interindividual heterogeneity influenced by several biological and environmental factors (Nyberg et al., 2020). Among the latter factors, structural and social determinants of health (SSDoH), defined as environmental conditions in which people live and that have an impact on health throughout life (Stites et al., 2021), play a significant role. Minority social identities (including race, ethnicity, sexual orientation, and gender identity) are considered to be SSDoH, as highlighted by the 2015 National Institute of Aging Health Disparities Research Framework proposed to advance knowledge on cognitive health disparities (Hill et al., 2015).

Health disparities in both self-reported and objectively assessed outcome measures have been long established in older ethnic and racial minority groups. In fact, Latino and Black American older adults, with and without dementia, generally report lower levels of quality of life than White Americans (Hayes-Larson et al., 2021). Negative social conditions, for example, discrimination, in older Black Americans have also been found associated with worse cognitive health, especially memory (Barnes et al., 2012), and greater brain damage, in particular, small hippocampal volume and greater white matter damage (Zahodne et al., 2023). The current literature on the risk factors for cognitive decline in older sexual (i.e., nonheterosexual people) and gender minorities (i.e., noncisgender people), instead, is much more limited compared with that of ethno-racial minorities. A possible explanation is that mental health, rather than cognitive health, has been the primary focus of investigations into lesbian, gay, bisexual, transgender, and other related minority (LGBT+) groups (Dai & Meyer, 2019). Currently, a few contrasting findings on cognitive performance and decline in sexual minority older adults have emerged: whereas this group was reported to have better verbal long-term memory than heterosexual older adults in two cohort studies (Manca et al., 2022; Stinchcombe & Hammond, 2021), a recent study has observed that people in same-sex relationships (SSR) had worse global cognitive performance and reached a diagnosis of cognitive impairment at a younger age than those in different-sex relationships (DSR; Hanes & Clouston, 2023). Additionally, the rate of decline in verbal memory has not been found associated with sexual orientation in a study by Stinchcombe and Hammond (2023). Similarly, no differences in rates of either mild cognitive impairment (MCI) or dementia diagnosis between people in SSR and DSR have been reported (Perales-Puchalt et al., 2019).

Investigations into the factors that may confer either higher risk for or protection against objectively assessed cognitive decline in sexual minority older adults are still lacking (Mielke et al., 2022). In fact, only a couple of studies have found that a higher risk of cognitive impairment in sexual minority older adult groups appears to be associated with greater depression severity (Hsieh et al., 2021) and higher likelihood to be not married (Liu et al., 2021). Correro and Nielson (2020) have proposed that cognitive health inequalities in sexual minority groups could also be explained as a possible consequence of minority stress (Meyer, 2003). The minority stress framework states that stigma and prejudice toward people with minority sexual orientations are linked to a series of specific stressors that are additional to the stress that people are exposed to ubiquitously (Meyer, 2003). This set of minority stressors has been traditionally divided into distal (i.e., objectively measurable sources of stress), such as structural stigma, discrimination, and various negative social experiences due to one’s sexual orientation, and proximal (i.e., subjective experiences of stress), such as internalized homophobia and expectations of stigma. Minority stress has been found to be associated particularly with worse mental (Bostwick et al., 2014) but also physical health (Caceres et al., 2017) in sexual and gender minorities. Both distal and proximal minority stressors have also been shown to affect several biological outcomes linked to a range of biological variables, for example, blood cell counts and cortisol levels (Flentje et al., 2020).

The literature on mental health inequalities due to minority stress is, by far, the most abundant and complementary theories that have been put forward to advance the understanding of the psychological mediators (Hatzenbuehler, 2009), the social-cognitive mechanisms and antecedents of minority stress, such as for example rejection sensitivity (Feinstein, 2020). Recent developments have also highlighted the potential impact of community-level, rather than interpersonal, factors that appear to have an impact on the health of sexual minority older adults (e.g., material deprivation). Indeed, nonheterosexual people have been found to earn less, in general, than heterosexual people, in particular, compared with heterosexual men (Waite & Denier, 2015). Discrimination related to sexual orientation appears to be a contributing factor. As a consequence, sexual minorities may be more likely to experience material deprivation (e.g., living in a deprived neighborhood), and this appears to have detrimental effects on their mental health (Yang et al., 2023).

The mechanisms behind health inequalities observed for sexual minority people are likely to be complex and possibly due to several interacting factors, including sexual orientation-related stigma (i.e., the core tenet of the minority stress model). However, investigations on the determinants of potential cognitive health in sexual minority older adults are lacking. To date, no studies have tested whether any proxy measures of minority stress are likely to be associated with either cognitive performance or cognitive decline in this population. Therefore, considering the limited knowledge about the factors that may affect cognition in sexual minority older adults, the aims of the present study were:

1) To compare cognitive performance between two groups of heterosexual and sexual minority older adults. It is hypothesized that the sexual minority group will show a more compromised cognitive profile than the heterosexual group, that is, worse cognitive performance;
2) To assess the differential impact of the risk factors of interest on cognitive performance in heterosexual and sexual minority older adults. It is hypothesized that all risk factors, in particular minority stress, will show stronger associations with worse cognitive performance in the sexual minority than in the heterosexual group;
3) To quantify the impact of the risk factors of interest on cognitive performance in the sexual minority group to enable direct comparisons with previous investigations. It is expected that all risk factors will be negatively associated with cognitive performance across cognitive measures.

## Research Design and Methods

### Participant Sample

This study used data from the English Longitudinal Study of Ageing (ELSA) data set available from the UK Data Archive, subject to registration (Banks et al., 2021). Over the course of 20 years, the ELSA has invited people aged 50 and over living in private households in England and their partners to be assessed every 2 years via self-completed questionnaires and face-to-face computer-assisted interviews. Ethical approval was obtained from the National Research Ethics Service and all participants gave full informed consent. Additionally, ethical approval for secondary data analysis included in this study was obtained from the College of Health, Medicine and Life Sciences Research Ethics Committee at Brunel University London.

The sample used in the present study has been identified by using data from Wave 6 (2012–2013), since, as part of this wave, a novel questionnaire was administered to collect information on sexual relationships and activities that were used to define participants’ sexual orientation. A total of 10,601 respondents were available at Wave 6. Participants were selected for this study if they met the following inclusion criteria: (1) aged 50 or older; (2) noninstitutionalized at the time of assessment; (3) availability of answers to the “sexual relationships and activities” questionnaire, in particular to the question on sexual desires (see section subsequently); (4) availability of sociodemographic characteristics (i.e., years of age, level of education, sex, and ethnicity); (5) no diagnosis of either dementia or of a neurodegenerative disease potentially leading to cognitive decline (i.e., Parkinson’s disease and Alzheimer’s disease). The final sample included 5,897 participants.

### Sexual Orientation

Self-reported sexual desires were used to define sexual orientation based on the answer provided to the question “*Which statement best describes your sexual desires over your lifetime?*.” Possible responses included: entirely for women; mostly for women, but some desires for men; equally for women and men; mostly for men, but some desires for women; entirely for men; and no sexual desires in a lifetime. Following an algorithm used by Grabovac et al. (2019), we excluded participants with no sexual desires over their lifetime and we divided the remaining participants as either heterosexual, that is, reporting sexual desires exclusively for people of the opposite sex, or sexual minority older adults, that is, reporting sexual desires for people of their same sex, either exclusively or to some degree. In the final sample, 94.3% of the participants (*n* = 5,561) were in the heterosexual group, while the sexual minority group represented 5.7% of the overall ELSA cohort (*n* = 336). The sexual minority group was not classified further into subgroups in order to avoid a reduction in statistical power.

### Risk Factors for Cognitive Decline

Self-reported depressive symptoms were quantified using the Center for Epidemiologic Studies Depression Scale (CES-D; Radloff, 1977). The CES-D includes eight items evaluating mood-related complaints in the past week that were scored using a dichotomous (*yes* = 1; *no* = 0) response, thus resulting in a total score between 0 and 8 (White et al., 2016).

Participants’ current marital status was coded as a binary variable to distinguish participants in a relationship from those who were not. At Wave 6, legal marital status was assessed by providing a series of response options: (1) Single, that is, never married; (2) Married, first and only marriage; (3) A civil partner in a legally recognized Civil Partnership; (4) Remarried, second, or later marriage; (5) Legally separated; (6) Divorced; and (7) Widowed. Due to small sample sizes across multiple categories, marital status was coded as a binary variable by distinguishing participants currently in a relationship (Options 2, 3, and 4) and those not in a relationship for multiple reasons (Options 1, 5, 6, and 7).

Minority stress was quantified using answers to two sets of questions on perceived discrimination asked as part of a paper self-completed questionnaire collected during Wave 5. The first set (“*In your day-to-day life, how often have any of the following things happened to you?*”) assessed whether participants reported experiences of everyday discrimination (i.e., stressful social experiences) based on a subset of those by Williams et al. (1997): (1) You are treated with less courtesy or respect than other people; (2) You receive poorer service than other people at restaurants or stores; (3) People act as if they think you are not clever; (4) You are threatened or harassed; (5) You receive poorer service or treatment than other people from doctors or hospitals. The second set (“*If any of the above things mentioned in the previous question have happened to you, what do you think were the reasons WHY these experiences happened to you?*”) assessed the reasons behind these experiences, that is, gender, race, age, weight, physical disability, an aspect of your physical appearance, sexual orientation, and financial status, based on the work by Kessler et al. (1999). A binary proxy measure of minority stress (experienced vs not experienced) was derived based on self-reported stressful experiences in different social contexts due to participants’ sexual orientation, in line with a previous study (Jackson et al., 2019). Among the 5,897 participants included in the study, 685 did not complete the interview at Wave 5, thus leaving 4,937 heterosexual and 275 sexual minority participants with data on minority stress.

### Cognitive Outcome Measures

Four cognitive measures were collected at Wave 6:

1) Orientation in time: a four-point measure assessing knowledge about the day of the week and the date (day, month, and year). Each question received a binary score (correct/incorrect) and the total score spanned between 0 and 4 (Smith et al., 2019);
2) Verbal long-term memory—learning: the Word list learning test developed by the United States-based Health and Retirement Study (HRS; Ofstedal et al., 2005) was used by presenting orally a list of 10 words to participants and then asking them to recall as many items as possible immediately after the reading (immediate recall, score 0–10);
3) Verbal long-term memory—retrieval: approximately after 5 min, during which participants completed other tasks, they were asked to recollect as many words as they could from the original list (delayed recall, score 0–10);
4) Fluid intelligence: assessed by means of the Number Series test (Fisher et al., 2013), a test developed to investigate quantitative reasoning in the HRS. In each trial, participants are given a series of numbers with one missing and to be identified based on the numerical pattern of the series. All participants complete a first block including three series of increasing difficulty. Subsequently, each participant is asked to complete another block (of three series) characterized by a level of difficulty calibrated based on the score obtained in the first block (0–3). The final score spanned between 0 and 15.

### Statistical Analysis

Demographic and cognitive risk factor profiles were compared between the sexual minority and the heterosexual groups by using the Mann–Whitney U test for continuous variables not normally distributed after inspection of the results of the Shapiro–Wilk test and the χ^2^ test for categorical variables.

To address Aim 1, general linear models were used to compare cognitive test scores between sexual orientation groups, by including years of age, educational qualification, and sex as covariates. The level of educational qualifications obtained by each participant has been coded using the eight-level category system used in England, Wales, and Northern Ireland (https://www.gov.uk/what-different-qualification-levels-mean/list-of-qualification-levels). Because within the ELSA study undergraduate, postgraduate, and doctoral degrees have not been differentiated, only one level was used for university-level education. As a result, a total of six education qualification levels were used: no qualifications; Level 1—lower General Certificate of Secondary Education (GCSE) grades; Level 2—higher GCSE grades; Level 3—A level; Level 4—higher certificates/vocational training; Level 5—higher diplomas/higher vocational training; and Levels 6/7/8—university degree/academic qualifications.

To address Aim 2, three general linear models including (1) sexual orientation, (2) each risk factor (i.e., depressive symptoms, marital status, and minority stress), (3) the interaction between sexual orientation and each risk factor, and (4) the same covariates used in the model to address the first aim were used to predict cognitive scores.

Finally, to investigate Aim 3, three models were used to assess the association between all cognitive test scores of the sexual minority group only and each risk factor by controlling for the same covariates used in the model to address the first aim.

All statistical analyses were carried out using IBM SPSS Statistics version 26 (IBM, Chicago, IL, USA). A significance threshold of *p* < .05 was used for all analyses. Because the data used in this study were collected as part of a survey, weighting was applied to correct for sampling probabilities and for differential nonresponse to the questionnaire on sexual relationships and activities by using the weights included in the Wave 6 data set of ELSA (NatCen Social Research, University College London, Institute for Fiscal Studies, 2023).

## Results

### Demographic Profiles

The sexual minority older adult group was younger and included a lower proportion of participants who were non-White and with lower educational levels (i.e., no qualifications and Level 1 qualifications), but a higher proportion of people with Level 4 qualifications than the heterosexual group (Table 1). Due to the very small sample size of the group of non-White sexual minority participants, this variable was not analyzed further but included as a complementary descriptor.

**Table 1.** Demographic Characteristics of the Sample (Displayed Figures are Weighted for Sampling Probabilities and Differential Nonresponse)

Variable	Heterosexual older adults (*n* = 5,561)^a^		Sexual minority older adults (*n* = 336)^a^		Test	*p* Value
	Median (Interquartile range)	%	Median (Interquartile range)	%		
Age (years)^b^	64.0 (15)		58.0 (14)		7.39	<.001
Sex^c^					3.48	.062
Male		48.8		43.7		
Female		51.2		56.3		
Ethnicity^c^					7.05	.008
White		94.9		98.0		
Non-White		5.1		2.2		
Education^c^						
No qualifications		**15.4**		**8.7**	36.11	<.001
Level 1		**9.5**		**6.5**		
Level 2		37.5		35.4		
Level 3		11.2		14.3		
Level 4		**14.3**		**23.6**		
Level 5		2.4		2.0		
Levels 6/7/8		9.7		9.5		

*Notes*: In bold: categories significantly different between groups.

^a^Unweighted sample sizes.

^b^Mann–Whitney U test (standardized test statistic).

^c^Chi-square test.

### Risk Factors for Cognitive Decline

When risk profiles were compared between groups (Table 2), no significant differences were found regarding depressive symptoms. Sexual minority participants were less likely to be in a relationship and more likely to report sexual orientation as the cause for such stressful experiences than the heterosexual group (5% vs 0.4% of respondents, respectively), although both groups reported similar rates of stressful experiences. However, similar high percentages of participants (43.4% for sexual minorities and 47.6% for heterosexual older adults) provided no answer to the question addressing sexual orientation as a potential cause of negative experiences.

**Table 2.** Risk Profiles Across Sexual Orientation Groups (Displayed Figures Are Weighted for Sampling Probabilities and Differential Nonresponse)

Variable	Heterosexual older adults (*n* = 5,561)^a^		Sexual minority older adults (*n* = 336)^a^		Test	*p* Value
	Median (Interquartile range)	%	Median (Interquartile range)	%		
Depressive symptoms						
CES-D^b^	1.0 (2)		1.0 (2)		−1.91	.056
Marital status						
Relationship^c^					35.52	<.001
Yes		67.5		52.1		
No		32.5		47.9		
Minority stress						
Stressful social experiences^c^^,^^d^						
Lack of respect					1.12	.572
Yes		54.3		57.7		
No		40.2		37.7		
Not answered		5.5		4.6		
Poor service in shops and restaurants					1.59	.451
Yes		37.6		41.8		
No		56.8		52.7		
Not answered		5.6		5.5		
Treated as less clever					2.11	.348
Yes		37.0		41.8		
No		57.4		53.2		
Not answered		5.6		5.0		
Threat/harassment					3.15	.207
Yes		24.3		29.5		
No		70.3		65.9		
Not answered		5.4		4.6		
Poor healthcare service					2.63	.268
Yes		15.5		19.5		
No		79.2		75.9		
Not answered		5.		4.6		
Sexual orientation as cause of stressful social experiences^c^^,^^d^					73.92	<.001
Yes		**0.4**		**5.0**		
No		52.0		51.6		
Not answered		47.6		43.4		

*Notes*: CES-D = Center for Epidemiologic Studies Depression Scale. In bold: specific categories significantly different between groups.

^a^Unweighted sample sizes.

^b^Mann–Whitney *U* test (standardized test statistic).

^c^Chi-square test.

^d^Unweighted sample sizes are *n* = 4,937 (heterosexual older adults) and *n* = 275 (sexual minority older adults).

### Cognitive Performance Differences Between Sexual Minority and Heterosexual Groups (Aim 1)

Table 3 shows that higher age, lower educational qualifications, and male sex were significantly associated with worse cognitive performance across all four tests. Sexual orientation, instead, was significantly associated with performance only on the delayed recall of the Word list learning test: the sexual minority group performed better than the heterosexual group (*B* = 0.283, *p* = .002), although the effect size was very small (Partial η^2^ = 0.001).

**Table 3.** General Linear Model Results of the Association Between Sexual Orientation and Demographic Characteristics and Cognitive Performance (Values Are Weighted for Sampling Probabilities and Differential Nonresponse)

Variable	*B* (*SE*)	95% CI	*p* Value	Partial η^2^
Orientation in time				
Minority sexual orientation	−0.020 (0.022)	−0.064, 0.024	.372	<0.001
Male sex	−0.047 (0.011)	−0.069, −0.026	<.001	0.003
No qualifications	−0.162 (0.023)	−0.207, −0.117	<.001	0.008
Education—L1	−0.077 (0.024)	−0.125, −0.029	.002	0.002
Education—L2	−0.097 (0.019)	−0.134, −0.060	<.001	0.004
Education—L3	−0.068 (0.023)	−0.112, −0.023	.003	0.001
Education—L4	−0.048 (0.022)	−0.090, −0.005	.027	0.001
Education—L5	−0.047 (0.039)	−0.122, 0.027	.210	<0.001
Age	−0.005 (0.001)	−0.006, −0.003	<.001	0.009
Word list—immediate recall				
Minority sexual orientation	0.103 (0.077)	−0.047, 0.253	.179	<0.001
Male sex	−0.492 (0.037)	−0.565, −0.419	<.001	0.026
No qualifications	−1.126 (0.078)	−1.278, −0.974	<.001	0.032
Education—L1	−0.705 (0.083)	−0.867, −0.542	<.001	0.011
Education—L2	−0.775 (0.064)	−0.901, −0.650	<.001	0.022
Education—L3	−0.391 (0.078)	−0.543, −0.239	<.001	0.004
Education—L4	−0.270 (0.073)	−0.414, −0.126	<.001	0.002
Education—L5	−0.380 (0.129)	−0.632, −0.127	.003	0.001
Age	−0.045 (0.002)	−0.049, −0.042	<.001	0.074
Word list—delayed recall				
Minority sexual orientation	**0.283 (0.093)**	**0.101, 0.466**	**.002**	**0.001**
Male sex	−0.581 (0.045)	−0.669, −0.492	<.001	0.025
No qualifications	−1.214 (0.095)	−1.400, −1.029	<.001	0.025
Education—L1	−0.811 (0.101)	−1.008, −0.613	<.001	0.010
Education—L2	−0.825 (0.078)	−0.978, −0.672	<.001	0.017
Education—L3	−0.544 (0.095)	−0.729, −0.358	<.001	0.005
Education—L4	−0.200 (0.089)	−0.375, 0.024	.025	0.001
Education—L5	−0.444 (0.157)	−0.752, −0.137	.005	0.001
Age	−0.054 (0.002)	−0.059, −0.050	<.001	0.071
Number Series test				
Minority sexual orientation	−0.063 (0.171)	−0.399, 0.273	.715	<0.001
Male sex	0.620 (0.083)	0.457, 0.784	<.001	0.008
No qualifications	−3.599 (0.174)	−3.940, −3.258	<.001	0.062
Education—L1	−1.927 (0.186)	−2.291, −1.563	<.001	0.016
Education—L2	−2.156 (0.144)	−2.438, −1.874	<.001	0.034
Education—L3	−0.983 (0.174)	−1.325, −0.642	<.001	0.005
Education—L4	−0.286 (0.165)	−0.608, 0.037	.082	<0.001
Education—L5	−1.815 (0.289)	−2.380, −1.249	<.001	0.006
Age	−0.046 (0.004)	−0.055, −0.037	<.001	0.016

*Notes*: B = general linear model coefficient; CI = Confidence interval; *SE* = Standard error.

Reference categories are: heterosexual older adults (for sexual orientation), female (for sex), Level 6 (for education).

Significant effects are highlighted in bold.

### Impact of Risk Factors on Cognitive Performance Across Sexual Orientation Groups (Aim 2)

Sexual minority participants performed reliably better than heterosexual older adults on the delayed recall of the Word list learning test across all models (Table 4). Moreover, minority sexual orientation was also associated with higher immediate recall scores, in models investigating depression and minority stress, and Number Series test scores, in the model including minority stress.

**Table 4.** General Linear Model Results of the Association Between Sexual Orientation, Risk Factors for Cognitive Decline and Cognitive Performance (Values are Weighted for Sampling Probabilities and Differential Nonresponse and Corrected for age, Education, and Sex)

Variable	*B* (*SE*)	95% CI	*p* Value	Partial η^2^
Depressive symptoms				
Orientation in time				
F1: Minority sexual orientation	−0.002 (0.030)	−0.060, 0.056	.936	<0.001
F2: CES-D	−**0.011 (0.003)**	−**0.018,** −**0.005**	**<.001**	**0.002**
F1 × F2 interaction	0.003 (0.012)	−0.020, 0.027	.774	<0.001
Word list—immediate recall				
F1: Minority sexual orientation	**0.200 (0.102)**	**0.001, 0.399**	**.049**	**0.001**
F2: CES-D	−**0.086 (0.011)**	−**0.107,** −**0.064**	**<.001**	**0.010**
F1× F2 interaction	−0.007 (0.041)	−0.088, 0.074	.866	<0.001
Word list—delayed recall				
F1: Minority sexual orientation	**0.400 (0.124)**	**0.157, 0.643**	**.001**	**0.002**
F2: CES-D	−**0.099 (0.014)**	−**0.126,** −**0.073**	**<.001**	**0.009**
F1 × F2 interaction	−0.002 (0.051)	−0.101, 0.097	.974	<0.001
Number series test				
F1: Minority sexual orientation	−0.152 (0.227)	−0.598, 0.294	.504	<0.001
F2: CES-D	−**0.215 (0.025)**	−**0.624,** −**0.166**	**<.001**	**0.013**
F1 × F2 interaction	0.145 (0.093)	−0.037, 0.326	.118	<0.001
Marital status				
Orientation in time				
F1: Minority sexual orientation	−0.007 (0.033)	−0.067, 0.053	.829	<0.001
F2: Marital status	0.006 (0.012)	−0.018, 0.030	.616	<0.001
F1 × F2 interaction	−0.031 (0.045)	−0.120, 0.057	.492	<0.001
Word list—immediate recall				
F1: Minority sexual orientation	0.199 (0.104)	−0.005, 0.402	.055	0.001
F2: Marital status	−**0.226 (0.041)**	−**0.307,** −**0.145**	**<.001**	**0.005**
F1 × F2 interaction	−0.119 (0.153)	−0.420, 0.181	.436	<0.001
Word list—delayed recall				
F1: Minority sexual orientation	**0.367 (0.126)**	**0.119, 0.614**	**.004**	**0.001**
F2: Marital status	−**0.284 (0.050)**	−**0.383,** −**0.186**	**<.001**	**0.005**
F1 × F2 interaction	−0.071 (0.002)	−0.437, 0.294	.702	<0.001
Number series test				
F1: Minority sexual orientation	−0.037 (0.232)	−0.492, 0.419	.874	<0.001
F2: Marital status	−**0.498 (0.093)**	−**0.680,** −**0.317**	**<.001**	**0.004**
F1 × F2 interaction	0.133 (0.343)	−0.540, 0.806	.699	<0.001
Minority stress				
Orientation in time				
* F1: Minority sexual orientation*	0.045 (0.039)	−0.031, 0.121	.247	0.001
* F2: SO-related stressful experiences*	−0.030 (0.104)	−0.233, 0.173	.775	<0.001
* F1 × F2 interaction*	−0.084 (0.165)	−0.407, 0.240	.613	<0.001
Word list—immediate recall				
* F1: Minority sexual orientation*	**0.459 (0.131)**	**0.202, 0.716**	**<.001**	**0.005**
* F2: SO-related stressful experiences*	−0.457 (0.351)	−1.145, 0.231	.193	0.001
* F1 × F2 interaction*	−0.056 (0.559)	−1.155, 1.039	.917	<0.001
Word list—delayed recall				
* F1: Minority sexual orientation*	**0.661 (0.161)**	**0.345, 0.978**	**<.001**	**0.006**
* F2: SO-related stressful experiences*	−0.095 (0.432)	−0.942, 0.752	.826	<0.001
* F1 × F2 interaction*	−0.832 (0.689)	−2.183, 0.519	.227	0.001
Number series test				
* F1: Minority sexual orientation*	**0.679 (0.297)**	**0.097, 1.260**	**.022**	**0.002**
* F2: SO-related stressful experiences*	−0.630 (0.794)	−2.186, 0.927	.428	<0.001
* F1 × F2 interaction*	−1.425 (1.266)	−3.908, 1.058	.260	<0.001

*Notes*: *B* = general linear model coefficient; CES-D = Center for Epidemiologic Studies Depression Scale; CI = confidence interval; F1 = first factor of interest; F2 = second factor of interest; *SE* = standard error; SO = sexual orientation.

Reference categories are: heterosexual older adults (for sexual orientation), being in a relationship (for marital status), and not having experienced stressful social experiences (for minority stress).

Significant effects are highlighted in bold.

In general, higher CES-D scores were negatively associated with cognitive performance on all tests, while not being in a relationship was associated with lower scores on all tests, except for orientation to time. Exposure to stressful experiences due to sexual orientation, instead, was not significantly associated with cognitive performance. Moreover, no significant interaction effects between sexual orientation and any of the three risk factors were observed.

### Impact of Risk Factors on Cognitive Performance in the Sexual Minority Group (Aim 3)

When the sexual minority group was investigated separately, a significant negative association was found between verbal long-term memory performance and both depression and marital status (Table 5). Moreover, a significant negative association between exposure to stressful experiences due to sexual orientation and performance on the Number Series test also emerged (*B* = −2.116, *p* = .016). Orientation in time performance, instead, was not significantly associated with any of the risk factors.

**Table 5.** General Linear Model Results of the Association Between Risk Factors for Cognitive Decline and Cognitive Performance in the Sexual Minority Older Adult Group (Values are Weighted for Sampling Probabilities and Differential Nonresponse and Corrected for Age, Education, and Sex)

Variable	*B* (*SE*)	95% CI	*p* Value	Partial η^2^
Depressive symptoms (CES-D)				
Orientation in time	−0.011 (0.012)	−0.034, 0.012	.336	0.002
Word list—immediate recall	−**0.121 (0.039)**	−**0.198,** −**0.044**	**.002**	**0.024**
Word list—delayed recall	−**0.120 (0.047)**	−**0.211,** −**0.028**	**.011**	**0.017**
Number series test	−0.145 (0.079)	−0.301, 0.011	.069	0.009
Marital status				
Orientation in time	−0.044 (0.047)	−0.137, 0.049	.348	0.002
Word list—immediate recall	−**0.358 (0.162)**	−**0.676,** −**0.040**	**.027**	**0.013**
Word list—delayed recall	−0.339 (0.191)	−0.715, 0.037	.077	0.008
Number series test	−0.282 (0.325)	−0.920, 0.357	.386	0.002
Minority stress (SO-related stressful experiences)				
Orientation in time	−0.199 (0.127)	−0.450, 0.052	.119	0.019
Word list—immediate recall	−0.309 (0.552)	−1.401, 0.783	.576	0.002
Word list—delayed recall	−0.612 (0.637)	−1.873, 0.648	.338	0.007
Number series test	−**2.116 (0.865)**	−**3.827,** −**0.404**	**.016**	**0.044**

*Notes*: *B* = general linear model coefficient; CES-D = Centre for Epidemiologic Studies Depression Scale; CI = confidence interval; *SE* = standard error; SO = sexual orientation.

Reference categories are: heterosexual older adults (for sexual orientation), being in a relationship (for marital status), and not having experienced stressful social experiences (for minority stress).

Significant effects are highlighted in bold.

## Discussion and Implications

In this study, the sexual minority group was over 12 times more likely to report stressful social experiences due to their sexual orientation than the heterosexual group (5% vs 0.4%), although both groups experienced similar levels of negative social experiences. This finding is consistent with the core tenet of the minority stress framework (Meyer, 2003) that identifies higher rates of sexual orientation-related stressors experienced by sexual minorities as a primary factor affecting health in this population. Moreover, sexual minority older adults were less likely to be in a relationship than heterosexual participants, in line with previous evidence (Fredriksen-Goldsen et al., 2017; Hsieh et al., 2021). Being either single or never married has been identified as a personal condition associated with worse cognition in sexual minority older adults in another cohort study (Hsieh et al., 2021). Despite these differences, however, both participant groups reported similar levels of depressive symptoms. This could be due to the fact that ELSA participants are generally healthy and that we excluded those affected by chronic neurological diseases. It must also be noted that a relatively small proportion of sexual minority older adults reported minority stress and, therefore, its impact may not have affected considerably this group.

Similarly, the sexual minority group had cognitive scores similar to those of the heterosexual participants but was found to have significantly better verbal long-term memory performance. This finding is consistent with previous cohort investigations on the Canadian Longitudinal Study on Aging (Stinchcombe & Hammond, 2021) and the United States-based National Alzheimer Coordinating Centre data sets (Manca et al., 2022). Despite the small effect size, this difference was reliably observed across statistical models accounting for between-group discrepancies in demographic characteristics and risk factors. The “healthy volunteer bias” (Lindsted et al., 1996) may explain this pattern of findings because it is possible that primarily sexual minority older adults who were very healthy and/or exposed to mild minority stressors decided to take part in the abovementioned studies. Moreover, better cognitive performance in the sexual minority group appears to be somehow against general predictions made by the minority stress framework (Correro & Nielson, 2020; Meyer, 2003) about worse health outcomes in LGBT+ than heterosexual people, when considered as whole groups. Indeed, differential exposure to minority stressors may drive heterogeneity in cognitive and other health outcomes in sexual minorities, as observed among younger gay and bisexual men living with HIV (Flentje et al., 2020).

For this reason, multiple risk factors for cognitive decline in sexual minority older adults have been investigated as potential predictors of cognitive performance. Although no interaction effects were found between these factors and sexual orientation, negative effects of depression severity and not being in a relationship emerged across most cognitive measures, particularly on verbal episodic memory in the sexual minority group. These findings confirm previous observations of negative associations between depression and a higher risk of worse global cognitive performance (Hsieh et al., 2021; Liu et al., 2021) and subjective cognitive complaints (Flatt et al., 2018) among sexual minorities and dementia in the general population (Rafnsson et al., 2020; Yu et al., 2020). Exposure to sexual orientation-related stressful experiences was also not associated with cognitive performance in the overall sample, possibly due to the low sample size of people reporting such risk factors. However, minority stress was a significant predictor of lower fluid intelligence scores in sexual minority older adults. To the best of our knowledge, this represents the first-ever source of evidence that a proxy measure of minority stress may capture a subgroup of sexual minority older adults who could be at a higher risk of cognitive decline. This result appears to be of particular relevance because it is consistent with the observation that the strongest impact of minority stress may be seen in sexual minority people aged 80 and older (Fredriksen-Goldsen et al., 2015). Moreover, it enriches a wealth of knowledge that has accumulated on the detrimental impact of various types of minority stressors and discrimination on cognitive health related to ethnicity and race (Barnes et al., 2012; Hayes-Larson et al., 2021) and weight (Sutin et al., 2019).

The first limitation of this study relates to the assessment of sexual orientation since no direct question was asked to ELSA participants at Wave 6 and, therefore, misclassification of a minor proportion of participants cannot be fully ruled out. Moreover, sexual orientation is commonly recognized as a multidimensional construct, including self-reported identity, sexual attraction/desire, and sexual behaviors. This means that focusing only on one of these dimensions may miss potential specific pathways of action of minority stress. Indeed, minority stress and, as a consequence, health inequalities may be expected to be significantly worse in people who self-identify as a sexual minority compared with individuals who only exhibit same-sex behaviors but who do not consider themselves as part of a sexual minority group. Although some findings do not fully support this hypothesis (McCabe et al., 2021), different mental (Bostwick et al., 2010) and physical health inequalities (Dyar et al., 2019) have been observed in sexual minority populations depending on the sexual orientation dimension measured. It is also possible that these highly correlated dimensions may interact with one another since higher odds of some chronic diseases have been found in self-identified sexual minority people who also had a history of same-sex sexual behaviors (Patterson & Jabson, 2018). Therefore, further investigations are needed to understand how minority stress may affect health inequalities in sexual minority older adults identified based on multiple sexual orientation dimensions. Second, the potential different impact of minority stress on sexual minority subgroups (e.g., gay/lesbian vs bisexual) was not investigated due to small sample sizes and, consequently, to prevent a loss of statistical power. However, this is a particularly important point to address in future studies, since different sexual minority groups may be exposed to variable levels of stigma, even originating from within the same minority group (Feinstein & Dyar, 2017). Third, this study was cross-sectional, as most studies in this field but one (Stinchcombe & Hammond, 2023), thus preventing any conclusions on the impact of minority stress and other risk factors on the trajectory of cognitive decline in sexual minority older adults over time. Longitudinal studies will be needed to investigate this issue that, to date, remains unexplored. Fourth, the proxy measure of minority stress used in this study was binary and only related to a limited set of social experiences, hence potentially failing to capture the dose-dependent effects of minority stress on cognitive outcomes. It must be mentioned, however, that previous studies using the same approach to assess minority stress in larger samples of sexual and gender minority people, found that those reporting perceived discrimination related to sexual orientation had a higher risk of depression, loneliness, and lower quality of life (Jackson et al., 2019; Sattler & Zeyen, 2021). Fifth, marital status was also coded as a binary variable, by distinguishing those participants for whom it was possible to determine that they were in a relationship (either married or in a civil partnership) from those who were not currently in a formally recognized relationship. As a result, the amount of sexual minority participants with a romantic partner might have been underestimated given that nonheterosexual people are less likely to be in a formally recognized relationship. However, previous studies have observed a positive effect of legal marital—rather than romantic relational—status on cognition both in sexual minority (Liu et al., 2021) and heterosexual older adult populations (Sundström et al., 2014). Finally, this study only investigated four cognitive measures available at Wave 6 of the ELSA data set and may have missed between-group differences in unexplored domains such as, for instance, attention, considering the results of a previous investigation on the general population of any age in England (Jacob et al., 2021). This limitation is common to all studies in this field that are all retrospective investigations of public data sets mostly focused on one cognitive outcome measure only (primarily episodic memory).

Therefore, the findings of this study suggest that the careful assessment of the history of minority stress in sexual minority older adults may offer insights for a more targeted clinical management of their health, including cognitive decline. Further research is needed to ascertain whether and how specific sexual minority subgroups may show a greater risk of cognitive decline across different domains (some still unexplored, such as social cognition) and whether this risk may potentially be associated with brain alterations, that, to date, have been explored only by two studies (Manca & Venneri, 2020; Manca et al., 2022). We argue that prospective investigations of selected samples should also be implemented to move knowledge in this field beyond that emerged so far from the exploitation of publicly available databases that all have several limitations (e.g., lack of data on sexual orientation, gender identity, and minority stress), and to test specific research hypotheses on the mechanisms that may foster or protect against cognitive decline in the aging sexual minority population.
